# Heedlessness in the Selection of Officers of Medical Societies

**Published:** 1907-05

**Authors:** 


### HEEDLESSNESS IN THE SELECTION OF OFFICERS OF MEDICAL SOCIETIES.

Like business corporations, the fate and prosperity of medical societies depend largely upon the efficiency of their officers. Unlike business corporations, medical societies are philanthropic organizations working toward ends for the advantage of the community. Selfish or personal interests have no place in them. The interests are strictly communal. It is doubly important, therefore, that the officers of such a society should represent the whole society and should be the most efficient members possibly obtainable for their positions. Many considerations enter into the determination of efficiency. For the sake of more concrete consideration, the first office, that of president, may be taken. The candidate for this office should be first of all a man of high ideals. He should be one who commands the respect of his profession. He should be capable as an executive officer, and ambitious for the advancement of the interests of medical science and practice through the instrumentality of his society. He should represent no coterie or faction, but should be equally interested in the whole society. If possible, he should be a man who can be agreed upon unanimously.

There are still other qualifications which contribute to the efficiency of such an officer. A man whose reputation extends beyond the confines of his county or State can add much to the prestige of his office, especially if he be known as a teacher or writer. In county societies a president who is widely and favorably known can be of especial value through his ability to secure eminent participants for his discussions. He can do much also to bring credit to his society in the eyes of the public. A certain degree of independence and leisure, out of which to give time and thought to his society is a desideratum. If the society have property interests, is erecting a building or developing a library, a man who can reach persons of means, who can interest philanthropists, and who is in touch with "the springs of affluence," may be able to serve his society in a most substantial manner. All of these things may be summed up in the word efficieyc. The efficient man is the one who serves to the best purpose.

The worst thing that can happen to a medical society is to elect officers for reasons other than that of efficiency. Some societies fall into the unfortunate practice of using the president's chair for the purpose of paying bills. They sell it to pay some officer who has served faithfully and well in one of the subordinate positions. To elect a secretary or librarian president of a society because he has placed the society under obligations to him for his services is quite equivalent to selling the office. The official who would accept or demand pay of this nature for his services would take from a society what means more than money to it. If the work of the subordinate officers is arduous, and the society can afford to pay them for their services, it may with propriety be done; but no society is so rich that it can afford to pay them with the president's chair in lieu of coin.

Many societies have what are called "time-honored customs"Jof always electing the president to a second term, or of re-electing the president to a second term, or of electing "our faithful secretary," or what not. There is room for only one custom, and that should be the custom of electing the best possible man available for the place, be he vice-president, secretary, treasurer, censor or non-official member from the working ranks.

Time-honored customs! Time honors no customs.—New York State Medical Journal.

